# Supplementary material for: Responses to conflicting binocular stimuli in mouse primary visual cortex
Source: bioRxiv. 2025 Sep 1:2024.12.31.630912. Originally published 2025 Jan 1. Preprint. [Version 2] doi: 10.1101/2024.12.31.630912 (PMC11722220; doi:10.1101/2024.12.31.630912)

bioRxiv preprint doi: https://doi.org/10.1101/2024.12.31.630912; this version posted September 1, 2025. The copyright holder has placed this preprint (which was not certified by peer review) in the Public Domain. It is no longer restricted by copyright. Anyone can legally share, reuse, remix, or adapt this material for any purpose without crediting the original authors.

Figure S1. Regular-spiking single unit responses to different stimulus conditions
Z-scored raster plots of regular-spiking (RS) single units for Monocular **(A)**, Concordant **(B)**, Phase Offset **(C)**, and Orthogonal **(D)** stimuli. Units were assigned to L2/3, L4, L5, or L6 based on the location of the electrode contact with the maximal single unit waveform, then sorted based on their average activity between 40-80ms in the Monocular condition. Differences between conditions and layers are quantified in Figure 5.

Figure S2. Fast-spiking single unit responses to different stimulus conditions
Same as Figure S3, but for fast-spiking (FS) single units.

Supplemental Table 1: Key Resources

**Reagent or resource**	**Source**	**Identifier**
Experimental models: organisms/strains		
Mouse: C57BL/6N	Bred in lab; Originally from Charles River	RRID:MGI:5651595
Mouse: Emx1-Cre	Bred in lab; Originally from Jackson	RRID:IMSR_JAX:005628
Mouse: Ai93(TITL-GCaMP6f)-D;CaMK2a-tTA	Bred in lab; Originally from Jackson	RRID:IMSR_JAX:024108
Mouse: SOM-IRES-Cre (C57BL/6N)	Bred in lab; Originally from Jackson	RRID:IMSR_JAX:018973
Software and algorithms		
Raw data	This paper	
MATLAB 2023b	Mathworks	RRID:SCR_001622
Prism 10	Graphpad	RRID:SCR_002798
Kilosort 2.5	Github	https://github.com/MouseLand/Kilosort
Phy	Github	https://github.com/cortex-lab/phy

bioRxiv preprint doi: https://doi.org/10.1101/2024.12.31.630912; this version posted September 1, 2025. The copyright holder has placed this preprint (which was not certified by peer review) in the Public Domain. It is no longer restricted by copyright. Anyone can legally share, reuse, remix, or adapt this material for any purpose without crediting the original authors.

VEPStimulusSuite: visual stimulus generation and presentation suite	Github (Jeff Gavornik, Boston University)	https://github.com/jeffgavornik/VEPStimulusSuite
Analyses were performed using custom-written MATLAB scripts	This paper	https://github.com/danielmontgomery7/suppression-analysis-code

1023

1024

Figure S1

bioRxiv preprint doi: https://doi.org/10.1101/2024.12.31.630912; this version posted September 1, 2025. The copyright holder has placed this preprint (which was not certified by peer review) in the Public Domain. It is no longer restricted by copyright. Anyone can legally share, reuse, remix, or adapt this material for any purpose without crediting the original authors.

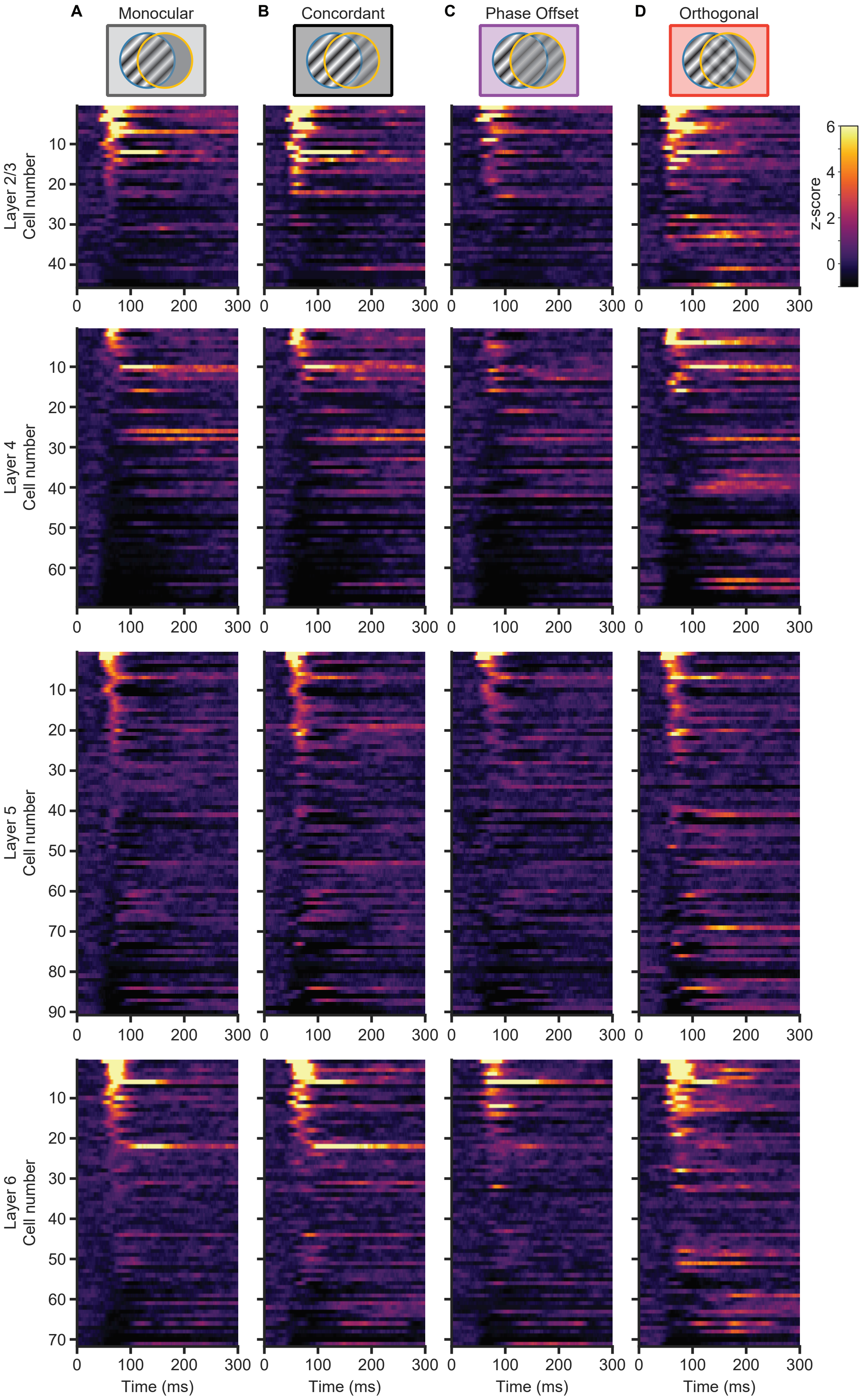

Figure S2

bioRxiv preprint doi: https://doi.org/10.1101/2024.12.31.630912; this version posted September 1, 2025. The copyright holder has placed this preprint (which was not certified by peer review) in the Public Domain. It is no longer restricted by copyright. Anyone can legally share, reuse, remix, or adapt this material for any purpose without crediting the original authors.

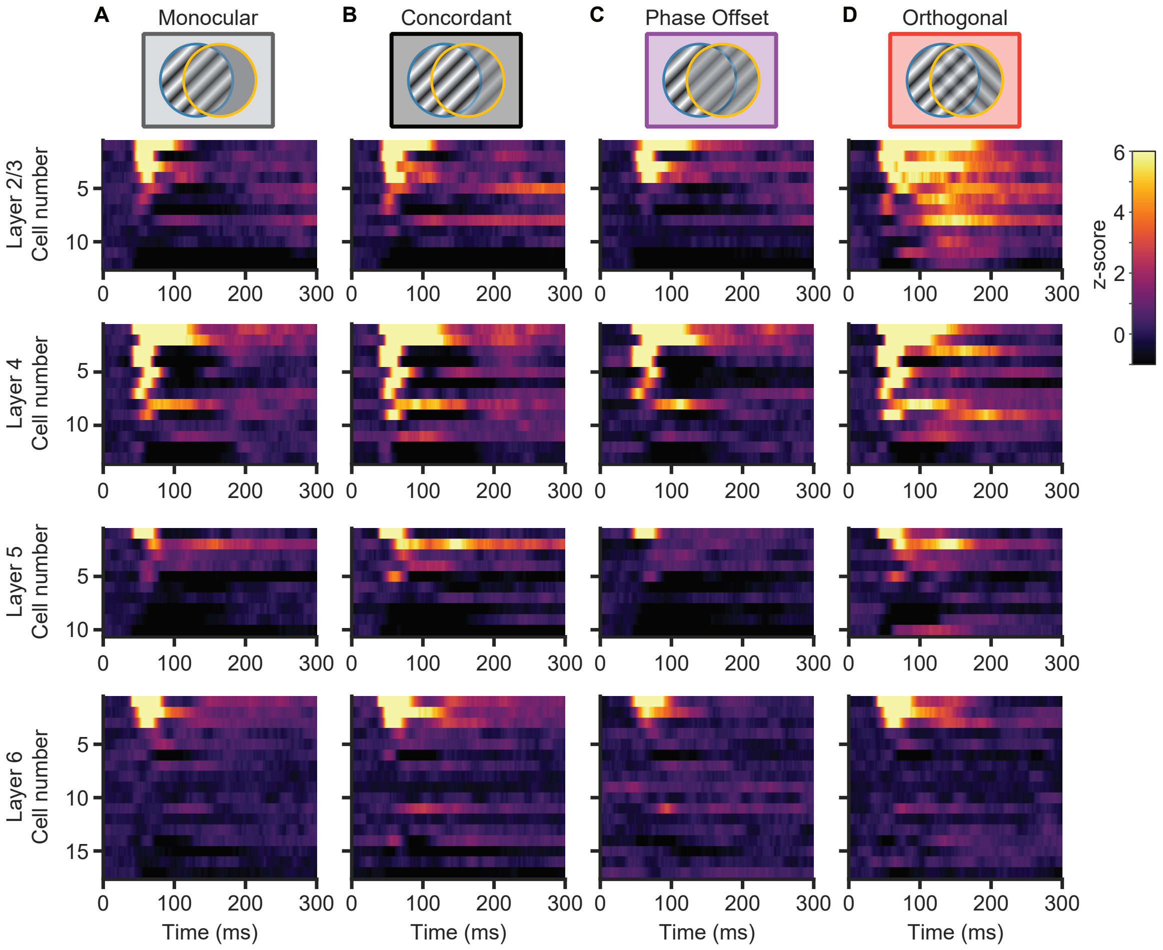

Supplement: Supplement 1 [file NIHPP2024.12.31.630912v2-supplement-1.pdf]
